# CCCTC-Binding Factor Locks Premature IgH Germline Transcription and Restrains Class Switch Recombination

**DOI:** 10.3389/fimmu.2017.01076

**Published:** 2017-09-04

**Authors:** Ester Marina-Zárate, Arantxa Pérez-García, Almudena R. Ramiro

**Affiliations:** ^1^B Lymphocyte Biology Laboratory, Fundacion Centro Nacional de Investigaciones Cardiovasculares Carlos III, Madrid, Spain

**Keywords:** class switch recombination, CCCTC-binding factor, germline transcription, activation-induced deaminase, somatic hypermutation

## Abstract

In response to antigenic stimulation B cells undergo class switch recombination (CSR) at the immunoglobulin heavy chain (IgH) to replace the primary IgM/IgD isotypes by IgG, IgE, or IgA. CSR is initiated by activation-induced cytidine deaminase (AID) through the deamination of cytosine residues at the switch (S) regions of IgH. B cell stimulation promotes germline transcription (GLT) of specific S regions, a necessary event prior to CSR because it facilitates AID access to S regions. Here, we show that CCCTC-binding factor (CTCF)-deficient mice are severely impaired in the generation of germinal center B cells and plasma cells after immunization *in vivo*, most likely due to impaired cell survival. Importantly, we find that CTCF-deficient B cells have an increased rate of CSR under various stimulation conditions *in vitro*. This effect is not secondary to altered cell proliferation or AID expression in CTCF-deficient cells. Instead, we find that CTCF-deficient B cells harbor an increased mutation frequency at switch regions, probably reflecting an increased accessibility of AID to IgH in the absence of CTCF. Moreover, CTCF deficiency triggers premature GLT of S regions in naïve B cells. Our results indicate that CTCF restricts CSR by enforcing GLT silencing and limiting AID access to IgH.

## Introduction

B cells remodel their immunoglobulin genes first as part of their differentiation program in the bone marrow, and then during the immune response, in the context of germinal centers (1). In the bone marrow, the heavy (IgH) and the light chain (IgL) immunoglobulin genes are subject to V(D)J recombination, a site specific recombination reaction triggered by RAG recombinases (2, 3). Subsequently, B cells that have been activated by antigen engage in the germinal center reaction where immunoglobulin genes are further diversified by somatic hypermutation (SHM) and class switch recombination (CSR), both of which are initiated by the activation-induced cytidine deaminase (AID) enzyme (4–6). SHM introduces (mostly) point mutations in the antigen recognizing, variable region of the IgH and IgL genes and antibody variants with higher affinity for antigen are selected through iterative rounds of mutation, proliferation, and cell death (7). CSR is a region-specific recombination reaction whereby the primary μ constant region (Cμ) is replaced by a downstream constant region (Cγ, Cε, or Cα) in the IgH gene, such that the primary IgM/IgD isotypes switch to IgG, IgE, or IgA (8, 9). Highly repetitive sequences called switch (S) regions preceding each constant region are targeted by AID, which triggers the deamination of cytosines followed by the generation of double strand breaks and the recombination reaction. CSR to a particular isotype is determined by stimulation-driven cues that activate specific S region I promoters, inducing their germline transcription (GLT) (10). GLT of both the donor and acceptor S regions is an absolute requirement for CSR, presumably by promoting transcription-coupled AID recruitment (11).

Both V(D)J recombination and CSR are tightly developmentally regulated, are intimately linked to transcription, and involve the formation of long-range DNA loops that facilitate the interaction between distant *cis* regulatory regions. CCCTC-binding factor (CTCF) is an architectural protein that regulates the genome function by mediating interactions between distant DNA sequences (12, 13) and is known to act as a transcriptional insulator through the generation of chromatin loops (14–16). CTCF regulates bone marrow B cell differentiation at different levels. First, CTCF-deficient pre-B cells show impaired proliferation and differentiation (17). Further, CTCF mediates interactions between distal and proximal regions of the IgH locus, regulating its transcription (18), and CTCF-binding elements (CBEs) are critical to inhibit rearrangement of proximal V_H_ genes and promoting rearrangement of distal V_H_ genes (19, 20). Finally, IgH employs CBE-based subdomains to regulate RAG on- and off-target activity (21). Regarding the role of CTCF during the germinal center reaction, we have recently shown that CTCF is an essential transcriptional regulator that allows high proliferation rate of germinal center B cells and represses the expression of Blimp-1, thus preventing plasma cell differentiation (22). However, the role of CTCF in CSR is not completely understood. The IgH locus is flanked by a 3′ regulatory region (3′RR), located 3′ of the most downstream C_H_, the Cα gene (23, 24). The proximal 3′RR contains several B cell-specific enhancers (hs3a, hs1,2, hs3b, and hs4) that are required for IgH GLT, class switching to all isotypes and high levels of Ig transcription in plasma cells (25–27). The distal 3′RR enhancer region (containing hs5, 6, 7, and 38 enhancers) is densely riveted with CBEs, and deletion of hs5–7 (harboring seven of the nine CBEs at the distal 3′RR) affected IgH locus compaction and V_H_ usage during V(D)J recombination, but did not affect CSR (28). In contrast, a CBE has been very recently identified within the Cα gene whose deletion promotes isotype-specific GLT in developing and resting B cells and alters CSR (29).

Here, we have directly assessed the role of CTCF in CSR using a conditional mouse model for CTCF depletion in B cells. We find that CTCF-deficient B cells show increased rate of CSR to various isotypes and under various stimulation conditions. This effect was not related to any measurable alterations of cell cycle or proliferation and was not a consequence of increased levels of AID expression. Instead, we find that AID activity is quantitatively increased on S regions. Finally, we show that GLT is markedly increased from activation-inducible, acceptor S regions in CTCF-deficient naïve B cells, indicating that CTCF restricts the transcription of activation-inducible S regions and limits AID accessibility and CSR.

## Materials and Methods

### Mice and Immunization

CCCTC-binding factor-deficient mice were obtained by breeding CTCF^fl/fl^ mice (30) with CD19-Cre^ki/+^ (31) mice. In all the experiments, CTCF^fl/+^; CD19-Cre^ki/+^ littermates were used as controls. Both males and females were used for experiments. All animals were housed in specific pathogen-free conditions, under a 12 h dark/light cycle with food *ad libitum*. All animal procedures were conducted according to EU Directive 2010/63/UE, enforced in Spanish law under Real Decreto 53/2013. The procedures have been reviewed by the Institutional Animal Care and Use Committee (IACUC) of Centro Nacional de Investigaciones Cardiovasculares and approved by Consejeria de Medio Ambiente, Administración Local y Ordenación del Territorio of Comunidad de Madrid (Ref: PROEX 341/14).

T-dependent immunization was induced in 6–11 weeks CTCF^fl/fl^; CD19-Cre^ki/+^ or CTCF^fl/+^; CD19-Cre^ki/+^ mice by intravenously injection of 10^8^ sheep red blood cells resuspended in 100 µl of sterile PBS. Immunization response was analyzed in spleen 7 days after sheep red blood cell injection. Number of animals per group to detect biologically significant effect sizes was calculated using appropriate statistical sample size formula and indicated in the biometrical planning section of the animal license submitted to the governing authority.

### Cell Cultures

Primary B cells from CTCF^fl/fl^; CD19-Cre^ki/+^ or CTCF^fl/+^; CD19-Cre^ki/+^ mice were isolated from spleen by immunomagnetic depletion using anti-CD43 beads (Miltenyi Biotec). Purified B cells were cultured at a final concentration of 1.2 × 10^6^ cell/ml in complete RPMI supplemented with 10% of FBS, 50 mM of 2-βmercaptoethanol (Gibco), 20 mM Hepes (Gibco) and 10 ng/ml of IL-4 (PeproTech) and 25 µg/ml of lipopolysaccharide (LPS, Sigma-Aldrich) or 1 µg/ml of anti-mouse CD40 (BD Pharmingen) and 10 ng/ml of IL-4 (PeproTech) to switch to IgG1; 25 µg/ml of LPS (Sigma-Aldrich) to switch to IgG3; 20 ng/ml of TGF-β (RD Systems), 1 µg/ml of anti-mouse CD40 (BD Pharmingen), and 10 ng/ml of IL-4 (PeproTech) to switch to IgG2b.

### Flow Cytometry

Single-cell suspensions were obtained from spleen or cultured B cells and stained with fluorophore-conjugated anti-mouse or human antibodies (BD Pharmingen or Invitrogen) to detect B220 (RA3-6B2, 1/200), Fas (Jo2, 1/400), GL7 (1/200), CD138 (281-2, 1/200), immunoglobulin G1 (IgG1, A85-1, 1/400), immunoglobulin G3 (IgG3, R40-82, 1/200), and immunoglobulin G2b (IgG2b, RMG2b-1, 1/200). Cell death was assessed by Annexin V (BD Pharmingen) staining. DNA incorporation was measured by BrdU staining (FITC BrdU Flow Kit, BD Pharmingen). Proliferation was assessed by cell trace CFSE staining (Invitrogen). Proliferation index was calculated as total number of divisions that took place in the culture divided by number of cells that have undergone at least one cell division. Samples were acquired on LSRFortessa or FACSCanto instruments (BD Biosciences) and analyzed with FlowJo Software.

### Next-Generation Sequencing

Analysis of Sμ mutations was performed as previously described (32). Briefly, DNA was extracted from LPS/IL-4 stimulated B cells from CTCF^fl/fl^; CD19-Cre^ki/+^ (*n* = 3) or CTCF^fl/+^; CD19-Cre^ki/+^ (*n* = 3) mice. Sμ fragment was PCR amplified using the oligonucleotides (forward) 5′-AAT GGA TAC CTC AGT GGT TTT TAA TGG TGG GTT TA-3′ and (reverse) 5′-GCG GCC CGG CTC ATT CCA GTT CAT TAC AG-3′. Amplification reactions were carried out with 2.5 U of Pfu Ultra (Stratagene) in a 50-μl reaction with the following profile: 94°C for 5 min followed by 25 cycles at 94°C for 10 s, 60°C for 30 s, and 72°C for 1 min. PCR products from four independent reactions per mouse were pooled and NGS sequencing was performed by the Genomics Unit at CNIC. Briefly, Libraries were prepared using NEBNext Ultra DNA Library Prep (New England Biolabs) following manufacturer’s instructions and sequenced on a HiSeq2500 (Illumina).

### qRT-PCR

RNA was extracted from CTCF^fl/fl^; CD19-Cre^ki/+^ or CTCF^fl/+^; CD19-Cre^ki/+^ mice using the Qiagen RNeasy kit and treated with DNAse. cDNA was synthesized using random hexamers (Roche) and SuperScript II reverse transcriptase. cDNA was quantified by SYBR green assay (Applied Biosystems) and normalized to GAPDH expression in duplicates. The following primers were used: mouse-GAPDH (forward) 5′-TGA AGC AGG CAT CTG AGG G-3′ (reverse) 5′-CGA AGG TGG AAG AGT GGG AG-3′; mouse-CTCF (forward) 5′-CAC CTG GGT CCT AAC AGA ACA GA-3′; mouse-CTCF (reverse) 5′-AGT ATG AGA GCG AAT GTG TCG TTT-3′, GLT-G1 (forward) 5′-TCG AGA AGC CTG AGG AAT GTG-3′; GLT-G1 (reverse) 5′-ATG GAG TTA GTT TGG GCA GCA-3′; GLT-G3 (forward) 5′-AGA GTC AGC CTC AAG GAG ATG AT-3′; GLT-G3 (reverse) 5′-CAG GGA CCA AGG GAT AGA CAG-3′, GLT-G2b (forward) 5′-CCA ACC AGG AAG AGT CCA GAG-3′; GLT-G3b (reverse) 5′-ACA GGG ATC CAG AGT TCC AAG T-3′; mouse-AID (forward) 5′-ACC TTC GCA ACA AGT CTG GCT-3′, mouse-AID (reverse) 5′-AGC CTT GCG GTC TTC ACA GAA-3′.

### Immunoblotting

B cells from LPS/IL-4 cultures were incubated on ice for 20 min in RIPA lysis buffer in the presence of protease inhibitors (Roche), and lysates were cleared by centrifugation. Total protein was size-fractionated on SDS-PAGE 12% acrylamide–bisacrylamide gels and transferred to Protran nitrocellulose membrane (Whatman) in transfer buffer (0.19 M glycine, 25 mM Tris base, and 0.01% SDS) containing 20% methanol (90 min at 0.4 A). Membranes were probed with anti-mouse-AID (1/25, eBioscience, 14-5959-82) and anti-mouse-MEK2 (1/2,000, BD Biosciences Pharmingen, 610,236). Then, membranes were incubated with HRP-conjugated anti-rat (1/5,000, Bethyl Laboratories, A110-105P) and anti-mouse (1/10,000, DAKO) antibodies, respectively, and developed with Clarity™ Western ECL Substrate (Bio-Rad).

### Statistics

Statistical analyses were performed with GraphPad Prism (version 6.01 for Windows, GraphPad Software, San Diego, CA, USA) using two-tailed Student’s *t*-test for all parameters conforming to normal distributions (according to Shapiro–Wilk normality test). Variance similarity was assessed with *F* test. *p* ≤ 0.05 was considered statistically significant. Error bars in figures represent SD.

## Results

**Figure 1:**
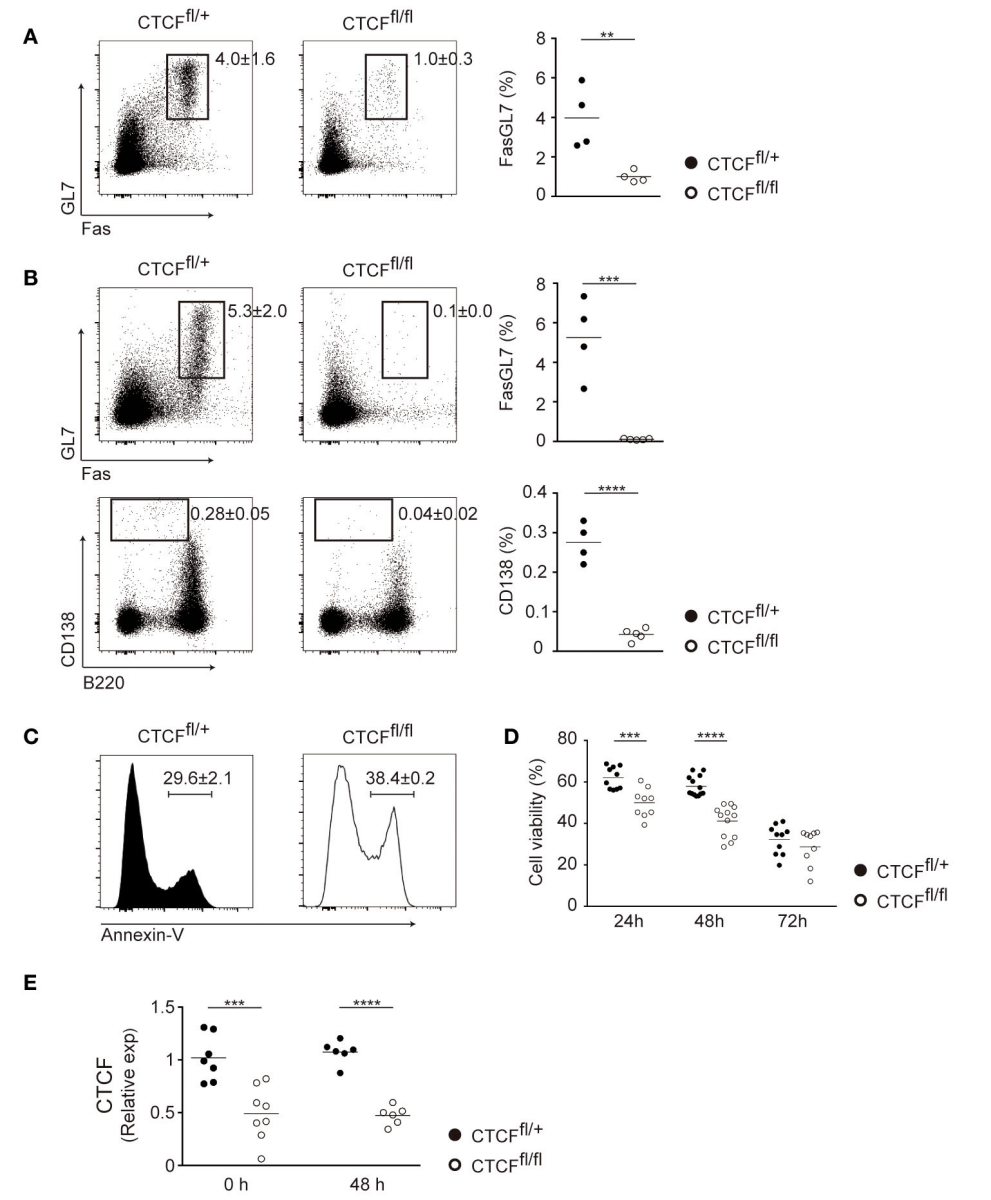
CCCTC-binding factor (CTCF) is required for the germinal center reaction in CTCF^fl/fl^; CD19-Cre^ki/+^ mice. **(A)** Flow cytometry analysis of Fas and GL7 expression in Peyer’s patch B cells from CTCF^fl/+^ (*n* = 4) and CTCF^fl/fl^ (*n* = 4) mice. Representative B220^+^ gated plots are shown on the left. Quantifications are shown on the right as percentage within B220^+^ cells. Each dot represents an individual mouse. *p*(Fas/GL7) = 0.01. **(B)** Flow cytometry analysis of GL7, Fas (top) and CD138, B220 (bottom) expression in splenic B cells from CTCF^fl/+^ (*n* = 4) and CTCF^fl/fl^ (*n* = 5) mice 7 days after SRBC immunization. Plots are gated on B220^+^ cells (top) or on total alive cells (bottom). Quantifications are shown on the right. Each dot represents an individual mouse. *p*(Fas/GL7) = 0.0006; *p*(B220/CD138) < 0.0001. **(C)** Annexin V staining in CTCF^fl/+^ (*n* = 2) and CTCF^fl/fl^ (*n* = 2) after 72 h of lipopolysaccharide (LPS) + IL-4 stimulation. A representative flow cytometry histogram is shown. Numbers indicate percentages ± SD. **(D)** Quantification of viable cells (7-AAD^−^) by flow cytometry analysis in CTCF^fl/+^ (*n* = 15) and CTCF^fl/fl^ (*n* = 14) after LPS + IL-4 stimulation; *p*(24 h) < 0.0001; *p*(48 h) < 0.0001. Each point corresponds to B cell cultures from an individual mouse. **(E)** qRT-PCR analysis of CTCF expression in resting and activated B cells from CTCF^fl/+^ (*n* = 7) and CTCF^fl/fl^ (*n* = 8) mice. *p*(0 h) = 0.0009; *p*(48 h) < 0.0001. Statistical analysis was done with two-tailed unpaired Student’s *t*-test.

To address the function of CTCF during CSR we generated a conditional mouse model to deplete CTCF late during B cell differentiation. CTCF^fl/fl^ mice (30) were bred to CD19-Cre^ki/+^, where the Cre recombinase is progressively expressed in the bone marrow and allows full depletion of floxed alleles at late stages of B cell differentiation (31). CTCF^fl/fl^; CD19-Cre^ki/+^ (CTCF-deficient mice, hereafter CTCF^fl/fl^) and CTCF^fl/+^; CD19-Cre^ki/+^ littermates (control mice, hereafter CTCF^fl/+^) were used in all experiments, since we have previously shown that CTCF is not haploinsufficient in mature B cells (22). We first analyzed the effect of CTCF depletion in Peyer’s patches, where germinal centers are constitutively generated in response to antigens of the gut microbiota, and found that CTCF^fl/fl^ Peyer’s patches are almost completely devoid of germinal center B cells (Figure 1A). To analyze the role of CTCF in *de novo* germinal center formation, we immunized CTCF^fl/fl^ and CTCF^fl/+^ mice with sheep red blood cells and analyzed spleen populations by flow cytometry 7 days after immunization. We found that both Fas^+^GL7^+^ germinal center B cells and CD138^+^ plasma cells were virtually absent in CTCF^fl/fl^ mice (Figure 1B), further indicating that CTCF is absolutely required for germinal center formation, and concomitantly, for plasma cell generation. *In vitro* stimulation of spleen B cells in the presence of LPS and IL-4 revealed that CTCF^fl/fl^ cells had a major B cell survival defect as measured by Annexin V staining (Figure 1C) and by viability stain exclusion (Figure 1D). Thus, these results suggest that CTCF is required for germinal center formation at least in part by enabling B cell survival.

We have previously generated a conditional mouse model where CTCF depletion is induced only upon AID expression [CTCF^fl/fl^; AID-Cre^TG/+^ mice (22)], i.e., delayed in comparison with the model presented here, where naïve B cells are already devoid of CTCF (Figure 1E). Consistently with the findings reported here, in CTCF^fl/fl^; AID-Cre^TG/+^ mice CTCF is also essential for the germinal center reaction (22). In contrast, B cells from the AID-Cre and the CD19-Cre models widely differ in their response to *in vitro* stimulation: while CTCF depletion in CTCF^fl/fl^; AID-Cre^TG/+^ B cells did not lead to any measurable defect upon CSR stimulation by LPS + IL-4 (22), CTCF^fl/fl^; CD19-Cre^ki/+^ B cells display a clear survival defect. Besides the functional implications of this disparity (discussed below), the CTCF^fl/fl^; CD19-Cre^ki/+^ model opens the possibility to study the function of CTCF in CSR, which could not be accurately addressed in CTCF^fl/fl^; AID-Cre^TG/+^ mice.

**Figure 2:**
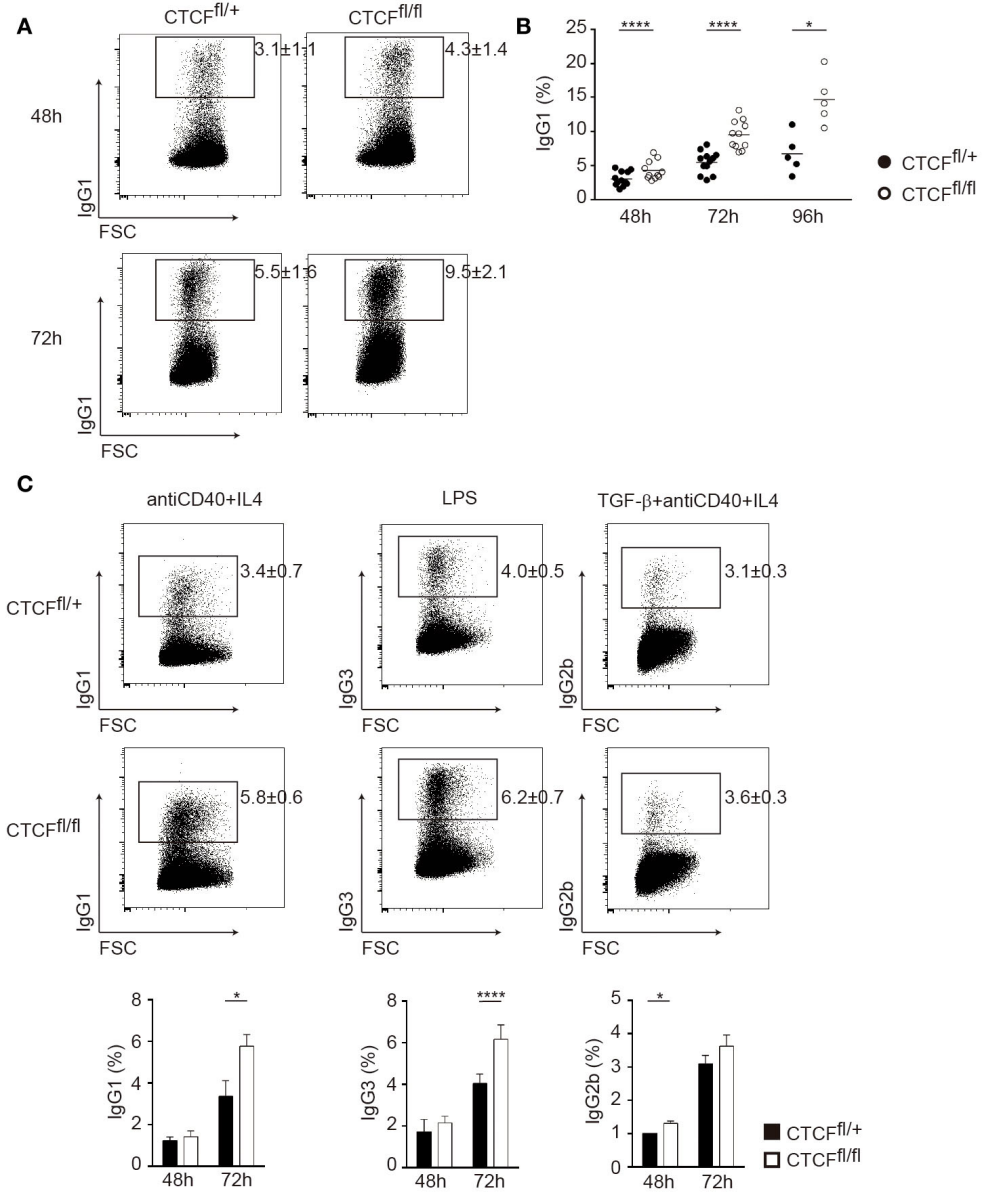
CCCTC-binding factor (CTCF) deficiency increases the rate of class switch recombination (CSR) *in vitro*. **(A)** Representative flow cytometry analysis of surface IgG1 expression 1 in splenic B cells from CTCF^fl/+^ and CTCF^fl/fl^ mice after 48 h (top panels) and 72 h (bottom panels) of lipopolysaccharide (LPS) + IL-4 stimulation. **(B)** Quantification of IgG1 cells in CTCF^fl/+^ (*n* = 12) and CTCF^fl/fl^ (*n* = 11) B cells. Each dot corresponds to a B cell culture from an individual mouse. Time of analysis is indicated underneath. Mean values are indicated in horizontal. *p*(48 h) < 0.0001; *p*(72 h) < 0.0001; *p*(96 h) = 0.0119. **(C)**. Representative flow cytometry analysis (top) of surface expression of CSR in splenic B cells from CTCF^fl/+^ (*n* = 4) and CTCF^fl/fl^ (*n* = 4) mice after 72 h of anti-CD40 + IL-4 (left), LPS (middle), and TGFβ + anti-CD40 + IL-4 (right) stimulation. Quantification of IgG 48 and 72 h after stimulation is shown on the bottom. *p*(anti-CD40 + IL-4) = 0.0022; *p*(LPS) = 0.0021; *p*(TGFβ + anti-CD40 + IL-4) = 0.033. Statistical analysis was done with two-tailed unpaired Student’s *t*-test.

To assess the role of CTCF in CSR we stimulated spleen B cells from CTCF^fl/fl^ and CTCF^fl/+^ mice *in vitro* in the presence of LPS and IL-4 and measured CSR to IgG1 by flow cytometry analysis. We found that CTCF-deficient cells consistently showed increased rate of CSR at all analyzed time points (Figures 2A,B). To determine whether the enhancement of CSR in CTCF-deficient cells was restricted to the IgG1 isotype or to the specific conditions of LPS + IL-4 stimulation, we stimulated CTCF^fl/fl^ and CTCF^fl/+^ B cells with anti-CD40 + IL-4, LPS, or TGFβ + anti-CD40 + IL-4, which preferentially trigger the activation of Iγ1, Iγ3, and Iγ2b S region promoters and the switch to IgG1, IgG3, and IgG2b, respectively. All three stimulations resulted in a notable increase in CSR in CTCF^fl/fl^ cells, indicating that CTCF deficiency broadly enhances the rate of CSR, regardless of the activation stimulus or the particular S region being used in the recombination reaction (Figure 2C). Thus, our results indicate that CTCF restricts the extent of CSR during B cell activation.

**Figure 3:**
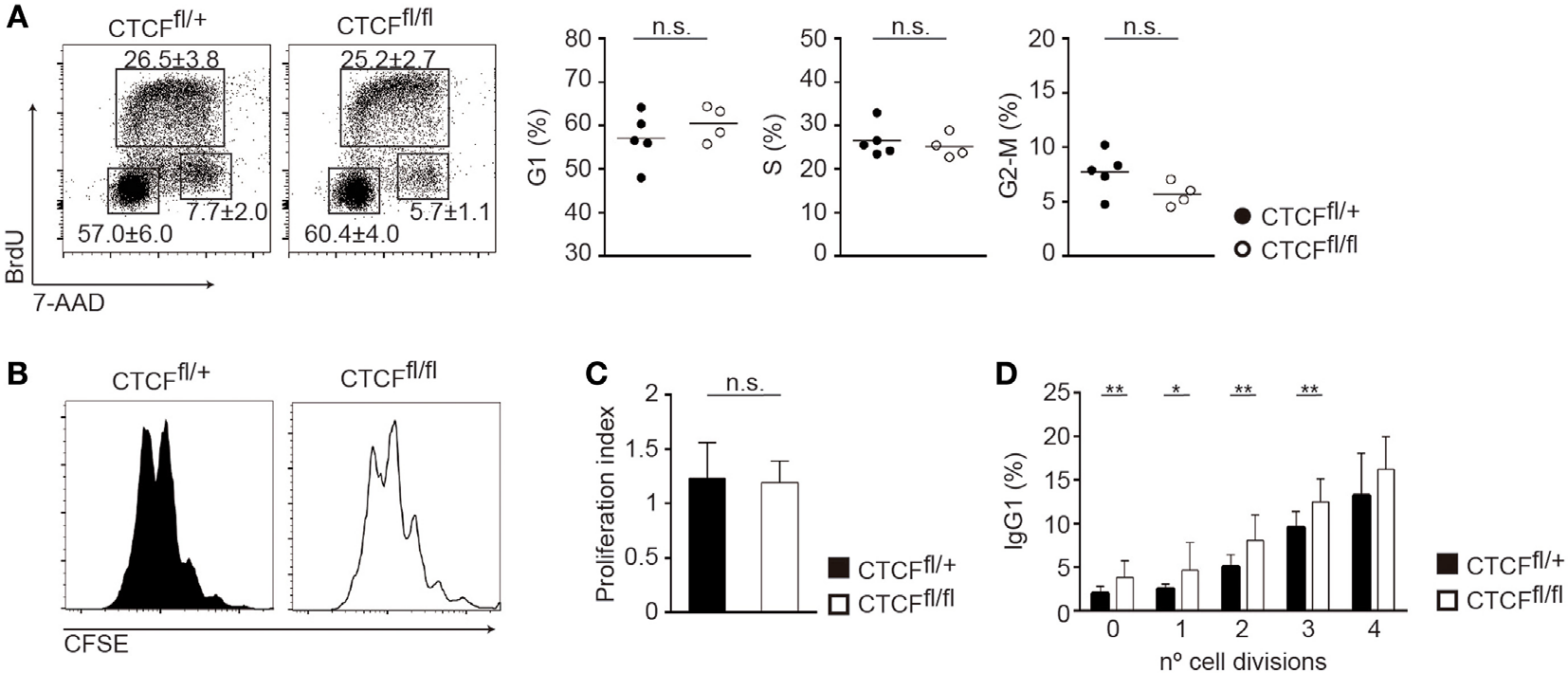
CCCTC-binding factor (CTCF) deficiency does not affect cell proliferation. **(A)** Cell cycle analysis of spleen B cells from CTCF^fl/+^ (*n* = 4) and CTCF^fl/fl^ (*n* = 4) mice 48 h after lipopolysaccharide (LPS) + IL-4 stimulation. DNA content was analyzed by flow cytometry after BrdU incorporation (30 min pulse) and 7-AAD staining. Representative plots are shown. Quantification of G1, S, and G2/M phase proportions is shown on the right. **(B)** CFSE staining of spleen B cells from CTCF^fl/+^ and CTCF^fl/fl^ mice 72 h after LPS + IL-4 stimulation. **(C)** Proliferation index of spleen B cells from CTCF^fl/+^ (*n* = 15) and CTCF^fl/fl^ (*n* = 14) mice 72 h after LPS + IL-4 stimulation measured by CFSE. **(D)** Percentage of IgG1 expression on each cell division measured by CFSE 72 h after LPS + IL-4 stimulation. *p*(0 division) = 0.0055; *p*(1 division) = 0.0298; *p*(2 division) = 0.0048; *p*(3 division) = 0.005. Numbers indicate percentages ± SD. Statistical analysis was done by two-tailed unpaired Student’s *t*-test.

Class switch recombination is tightly associated with proliferation (9, 33), such that B cells that have undergone more cell divisions accumulate a bigger fraction of isotype-switched cells. To investigate whether the increased rate of CSR could be secondary to an alteration in cell proliferation, we first performed cell cycle analysis of CTCF^fl/fl^ and CTCF^fl/+^ B cells stimulated with LPS + IL-4. We could not detect any alteration in the proportion of cells in G1, S, or G2/M as measured by BrdU incorporation (Figure 3A). The rate of cell proliferation analyzed by CFSE dilution and the proliferation index were also indistinguishable between CTCF^fl/fl^ and CTCF^fl/+^ LPS + IL-4 stimulated B cells (Figures 3B,C). Further, we measured the rate of CSR to IgG1 as a function of the number of cell divisions, and we found that CTCF^fl/fl^ cells display an enhanced CSR frequency compared to CFCF^fl/+^ cells, regardless of the number of divisions they have undergone (Figure 3D). Together, these observations indicate that the increased CSR rate observed in CTCF-deficient B cells is not a collateral effect of cell proliferation alterations and instead it suggests that it could be intrinsically related to the molecular mechanism of CSR.

**Figure 4:**
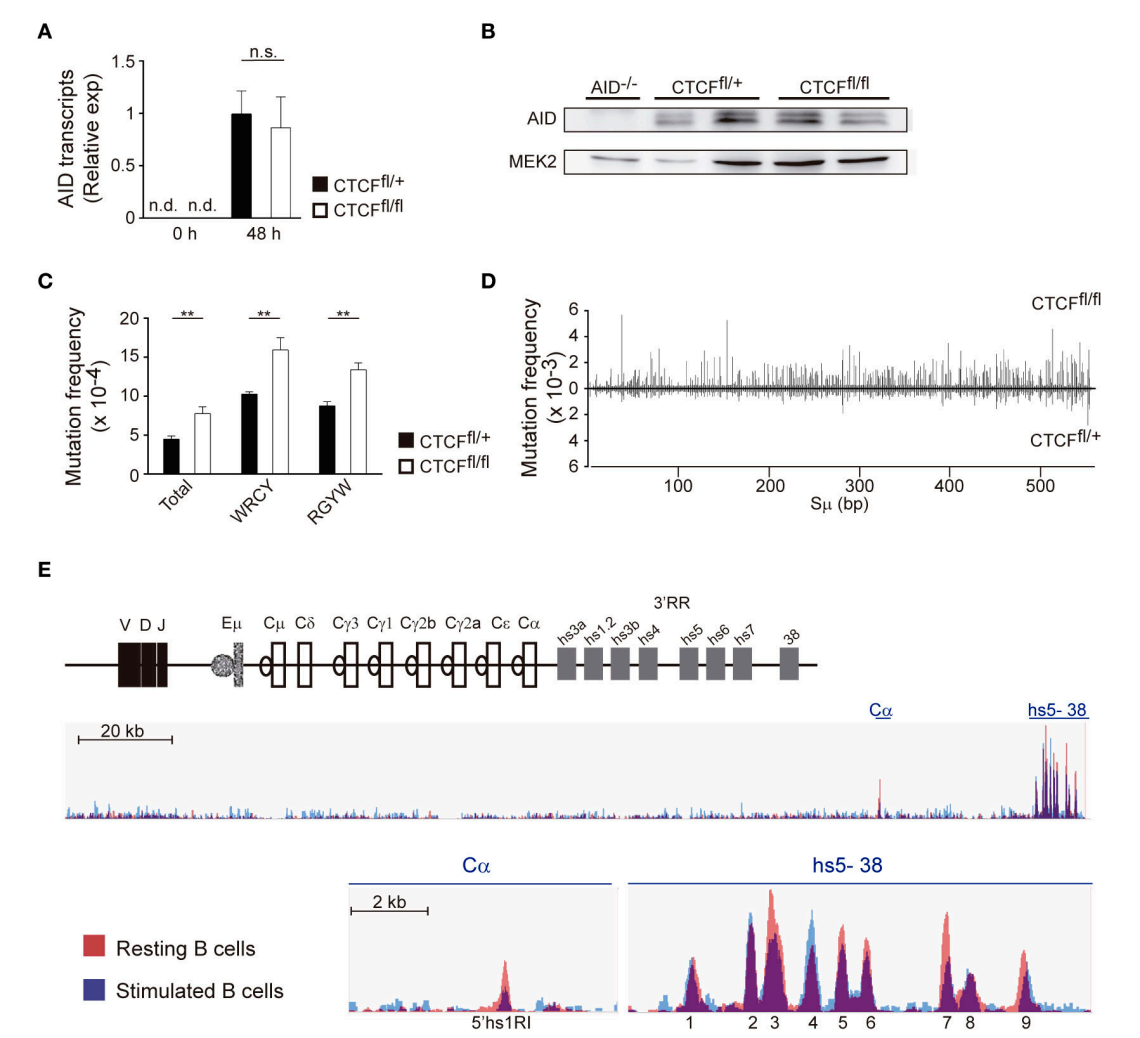
CCCTC-binding factor (CTCF) deletion increases S region somatic mutation and germline transcription. **(A)** qRT-PCR analysis of activation-induced cytidine deaminase (AID) expression in spleen B cells from CTCF^fl/+^ (*n* = 7) and CTCF^fl/fl^ (*n* = 6) mice 48 h after lipopolysaccharide (LPS) + IL-4 stimulation. **(B)** Western blot analysis of AID in B cells from AID^−/−^, CTCF^fl/+^, and CTCF^fl/fl^ mice after 48 h of LPS/IL-4 stimulation. Mek2 is shown as a loading control. **(C)** Analysis of AID mutagenic activity by next-generation sequencing (32). DNA was isolated from splenic B cells 48 h after LPS + IL-4 stimulation, amplified by PCR with specific primers and analyzed by next-generation sequencing. Graphs show total mutation frequency, mutation frequency at C or G in WRCY/RGYW hotspot motif in CTCF^fl/+^ (*n* = 3) and CTCF^fl/fl^ (*n* = 3) mice. *p*(total) = 0.0039; *p*(WRCY) = 0.0037; *p*(RGYW) = 0.0015. **(D)** Distribution of mutations along the sequenced Sμ region in CTCF^fl/+^ (*n* = 3) and CTCF^fl/fl^ (*n* = 3) mice. Mutation frequency at each position is shown. **(E)** CTCF-binding at the IgH locus in resting and stimulated B cells (data from GSE43594).

Activation-induced cytidine deaminase initiates CSR by deaminating cytosines on the IgH S regions, and AID levels roughly correlate with the efficiency of CSR (34). To address whether CTCF regulates the expression of AID during B cell activation, CTCF^fl/fl^ and CTCF^fl/+^ B cells were stimulated with LPS + IL-4, and AID levels were assessed by qRT-PCR and western blot analysis. We found that CTCF deficiency did not significantly affect the expression of AID at the mRNA or protein level (Figures 4A,B).

A fraction of AID-induced deaminations on S regions are processed into DNA double strand breaks, while others give rise to mutations that can be used to track AID activity (33, 35–37). Thus, we next compared the mutation frequency in Sμ of CTCF^fl/fl^ and CTCF^fl/+^ B cells stimulated with LPS + IL-4 by PCR-Seq (32). We found that Sμ regions of CTCF^fl/fl^ B cells accumulated a significantly higher frequency of mutations than CTCF^fl/+^ B cells (Figure 4C). In both CTCF-proficient and -deficient B cells, mutations clustered preferentially at WRCY/RGYW AID mutational hotspots (38) (Figure 4C) and extended throughout the length of the sequenced region (Figure 4D; Table S1 in Supplementary Material). These findings indicate that AID-induced mutation frequency at S regions is quantitatively increased in CTCF^fl/fl^ B cells, suggesting that CTCF deficiency could enhance AID activity by facilitating its access to S regions at IgH. In agreement with this idea, we found that the density of CTCF-binding to the different CBEs interspersed at the IgH locus was altered in LPS/IL-4 stimulated B cells as compared with naïve B cells (39) (Figure 4E). Specifically, we found that several CBEs, such as the 5′hs1R1 site (29, 39), and peaks 3 and 7 at the 3′RR (Figure 4E) were more densely bound by CTCF in resting than in activated B cells, while we found no difference or the reverse trend for other CBEs. This analysis indicates that during B cell activation, binding of CTCF to the IgH is rearranged, suggesting that CTCF could be an important factor involved in chromatin remodeling of IgH during B cell activation.

**Figure 5:**
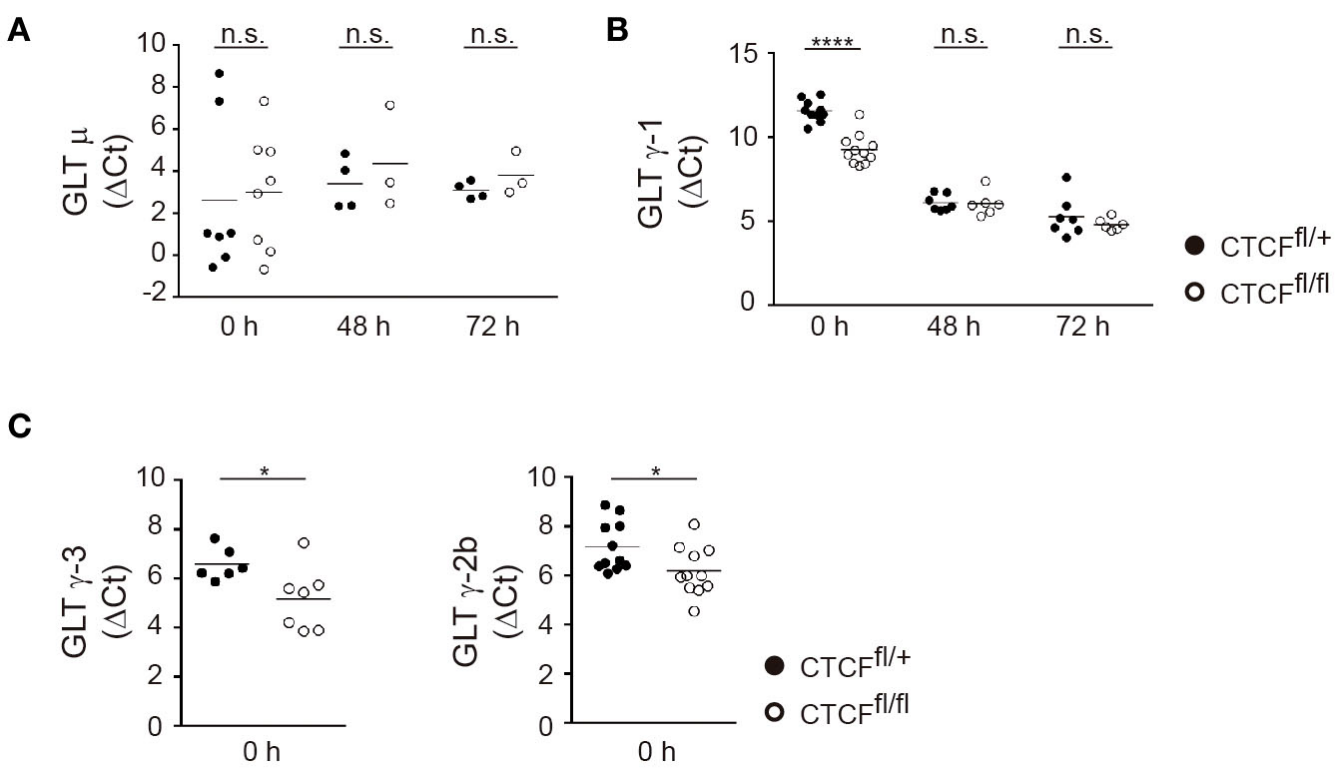
CCCTC-binding factor (CTCF) deficiency enhances germline transcription (GLT) in resting B cells. **(A,B)** qRT-PCR analysis of germline transcript μ **(A)**, γ-1 **(B)** in resting splenic B cells (0 h) or after lipopolysaccharide + IL-4 stimulation from CTCF^fl/+^ and CTCF^fl/fl^ (*n* = 3–7). *p*(0 h, GLTγ1) < 0.0001. **(C)** qRT-PCR analysis of germline transcript γ-3 (left), γ-2b (right) in resting splenic B cells. *p*(0 h, GLTγ3) = 0.0353; *p*(0 h, GLTγ2b) = 0.0307. Data are shown as the ΔCt value calculated from the difference in the Ct of the GLT and GAPDH. Statistical analysis was done with two-tailed unpaired Student’s *t*-test.

To gain further insights into the role in CTCF in regulating IgH accessibility, we measured GLT at donor and acceptor S regions in CTCF-proficient and -deficient B cells. GLT is absolutely required for CSR and is widely considered an indicator of S region accessibility (10, 11). While Sμ is transcribed constitutively, GLT at acceptor S regions is induced only upon appropriate stimulation of I promoters. We measured GLT at S regions in naïve and stimulated B cells and found that Sμ transcription was not affected in CTCF^fl/fl^ B cells compared to CTCF^fl/+^ B cells, regardless of their resting or activated state (Figure 5A). In contrast, GLT expression of Sγ1 acceptor region was significantly increased in naïve B cells, but not in activated B cells (Figure 5B). Accordingly, GLT-γ3 and GLT-γ2b also showed a clear increase in CTCF^fl/fl^ naïve B cells when compared to CTCF^fl/+^ B cells (Figure 5C). Together, these results show that CTCF prevents GLT of S regions prior to B cell activation, and that CTCF deficiency unleashes transcription from the I promoters in naïve B cells.

## Discussion

In this work, we have assessed the role of CTCF in CSR making use of a conditional mouse model for CTCF depletion in mature B cells (CTCF^fl/fl^; CD19-Cre^ki/+^). We previously explored the function of CTCF in germinal center B cells using a different mouse model (CTCF^fl/fl^; AID-Cre^TG/+^), which promotes CTCF depletion only after B cell activation and induction of AID expression (22). Thus, in the case of CTCF^fl/fl^; CD19-Cre^ki/+^ mice, the levels of CTCF are dramatically reduced already in naïve B cells (Figure 1E), while in CTCF^fl/fl^; AID-Cre^TG/+^ mice CTCF depletion is delayed and dependent on B cell activation and thus naïve B cells have normal CTCF expression (22). Interestingly, these mouse models have revealed important functional differences upon B cell activation. CTCF^fl/fl^; AID-Cre^TG/+^ B cells stimulated in the presence of LPS and IL-4 do not have a detectable phenotype, while CTCF^fl/fl^; CD19-Cre^ki/+^ B cells show a compromised cell viability and an increase in CSR. This phenotype is supported by the finding that under these conditions, the transcriptome of CTCF^fl/fl^; AID-Cre^TG/+^ B cells are almost intact (22), while LPS/IL-4 stimulated CTCF^fl/fl^; CD19-Cre^ki/+^ B cells show drastic transcriptome alterations (our unpublished data). Thus, on one hand, these alternative genetic models have unveiled distinct contributions of CTCF at early and late stages of B cell activation, which will be further investigated. On the other hand, the CTCF^fl/fl^; CD19-Cre^ki/+^ model has established an important role of CTCF in the regulation of CSR, the focus of this study.

We have found here that CTCF depletion in CTCF^fl/fl^; CD19-Cre^ki/+^ B cells increases the rate of CSR to all isotypes tested, and it does so without significantly altering B cell proliferation rate. This is an interesting observation because it is a first hint that, regardless of the potentially broad functional consequences of CTCF depletion, CTCF^fl/fl^; CD19-Cre^ki/+^ B cells may have an intrinsic CSR defect. In search for this defect we first assessed AID levels and found that CTCF has not a noticeable role in the regulation of AID expression. In contrast, we found that the mutation frequency at Sμ is significantly increased in the CTCF^fl/fl^; CD19-Cre^ki/+^ B cells, suggesting that the accessibility of AID to the switch regions is enhanced in the absence of CTCF. In agreement with this hypothesis, we find that B cell activation results in a redistribution of CTCF-binding along the IgH locus, and that GLT is increased in CTCF-deficient naïve B cells.

Although we cannot rule out that CTCF could also restrict CSR by the transcriptional silencing of factors, other than AID, important to trigger GLT or to process AID-induced lesions, our data strongly suggest that CTCF restricts GLT and CSR by its interaction with CBEs found in the IgH locus. In this regard, our findings are reminiscent of recent data that made use of a complementary approach (29). Specifically, Khamlichi and coworkers generated mice with a germline deletion of a DNAseI hypersensitivity site along with a CBE (5′hs1R1) and found activation of GLT at various S regions in naïve B cells and isotype-specific alterations of CSR after B cell activation. These data suggest that 5′hs1R1 contains a CBE acting as a CTCF insulator that precludes premature activation of I promoters in S regions (29). Likewise, our data strongly suggest that CTCF binds to CBEs at IgH, preventing GLT prior to B cell stimulation. Dissecting the contribution of specific CBEs and CTCF-mediated chromatin loops to particular CSR phenotypic effects is an essential issue remaining. First, it will be important to precisely define the CTCF-dependent loops taking place before and after B cell activation by 3C—as previously described for CSR synapsis (40). This should be complemented with functional analysis performing individual and combined deletion of CBEs; this will prove a very challenging task, given the complex array of CBEs at the constant region of IgH—including at least the 5′hs1R1 CBE and nine CBEs downstream of the 3′RR.

The finding that CTCF depletion promotes increase of GLT in resting but not activated B cells is intriguing. We speculate that CTCF contributes to establish a chromatin architecture that keeps the I promoters silent prior to CSR. Unleashing this architectural constraint in naïve B cells by depleting CTCF may facilitate the early loading or pre-poising of CSR cofactors that could allow faster or better loading of AID and additional CSR components at the time of activation. Although this hypothesis would require experimental testing, it agrees with previous observations made upon depletion of the 5′hs1R1 CBE (29).

In sum, our results are compatible with a model where CTCF maintains the IgH locus in a locked architectural conformation in naïve B cells that blocks GLT and AID access to S regions, thus preventing premature CSR.

## Ethics Statement

All animal procedures were conducted according to EU Directive 2010/63/UE, enforced in Spanish law under Real Decreto 53/2013. The procedures have been reviewed by the IACUC of Centro Nacional de Investigaciones Cardiovasculares and approved by Consejeria de Medio Ambiente, Administración Local y Ordenación del Territorio of Comunidad de Madrid (Ref: PROEX 341/14).

## Author Contributions

EM-Z and AP-G designed experiments, performed experiments, and wrote manuscript. AR designed experiments and wrote the manuscript.

## Conflict of Interest Statement

The authors declare that the research was conducted in the absence of any commercial or financial relationships that could be construed as a potential conflict of interest.

## Supplementary Material

Table S1 The mutation analysis of the Sμ region CTCF^fl/+^ and CTCF^fl/fl^ B 48h after lipopolysaccharide + IL-4 stimulation.

## References

[1] Rajewsky K. Clonal selection and learning in the antibody system. Nature (1996) 381:751–8. 10.1038/381751a0 8657279

[2] Jung D Giallourakis C Mostoslavsky R Alt FW. Mechanism and control of V(D)J recombination at the immunoglobulin heavy chain locus. Annu Rev Immunol (2006) 24:541–70. 10.1146/annurev.immunol.23.021704.115830 16551259

[3] Schatz DG Ji Y. Recombination centres and the orchestration of V(D)J recombination. Nat Rev Immunol (2011) 11:251–63. 10.1038/nri2941 21394103

[4] Casellas R Basu U Yewdell WT Chaudhuri J Robbiani DF Di Noia JM. Mutations, kataegis and translocations in B cells: understanding AID promiscuous activity. Nat Rev Immunol (2016) 16:164–76. 10.1038/nri.2016.2 26898111 PMC4871114

[5] Di Noia JM Neuberger MS. Molecular mechanisms of antibody somatic hypermutation. Annu Rev Biochem (2007) 76:1–22. 10.1146/annurev.biochem.76.061705.090740 17328676

[6] Muramatsu M Kinoshita K Fagarasan S Yamada S Shinkai Y Honjo T. Class switch recombination and hypermutation require activation-induced cytidine deaminase (AID), a potential RNA editing enzyme. Cell (2000) 102:553–63. 10.1016/S0092-8674(00)00078-7 11007474

[7] Victora GD Nussenzweig MC. Germinal centers. Annu Rev Immunol (2012) 30:429–57. 10.1146/annurev-immunol-020711-075032 22224772

[8] Matthews AJ Zheng S DiMenna LJ Chaudhuri J Regulation of immunoglobulin class-switch recombination: choreography of noncoding transcription, targeted DNA deamination, and long-range DNA repair. Adv Immunol (2014) 122:1–57. 10.1016/B978-0-12-800267-4.00001-8 24507154 PMC4150736

[9] Stavnezer J Guikema JE Schrader CE. Mechanism and regulation of class switch recombination. Annu Rev Immunol (2008) 26:261–92. 10.1146/annurev.immunol.26.021607.090248 18370922 PMC2707252

[10] Matthews AJ Zheng S DiMenna LJ Chaudhuri J Regulation of immunoglobulin class-switch recombination. Adv Immunol (2014) 122:1–57. 10.1016/B978-0-12-800267-4.00001-8 24507154 PMC4150736

[11] Pavri R Nussenzweig MC. AID targeting in antibody diversity. Adv Immunol (2011) 110:1–26. 10.1016/B978-0-12-387663-8.00005-3 21762814

[12] Ong CT Corces VG. CTCF: an architectural protein bridging genome topology and function. Nat Rev Genet (2014) 15:234–46. 10.1038/nrg3663 24614316 PMC4610363

[13] Merkenschlager M Odom DT. CTCF and cohesin: linking gene regulatory elements with their targets. Cell (2013) 152:1285–97. 10.1016/j.cell.2013.02.029 23498937

[14] Hnisz D Day DS Young RA. Insulated neighborhoods: structural and functional units of mammalian gene control. Cell (2016) 167:1188–200. 10.1016/j.cell.2016.10.024 27863240 PMC5125522

[15] Majumder P Gomez JA Chadwick BP Boss JM. The insulator factor CTCF controls MHC class II gene expression and is required for the formation of long-distance chromatin interactions. J Exp Med (2008) 205:785–98. 10.1084/jem.20071843 18347100 PMC2292219

[16] Splinter E Heath H Kooren J Palstra RJ Klous P Grosveld F CTCF mediates long-range chromatin looping and local histone modification in the beta-globin locus. Genes Dev (2006) 20:2349–54. 10.1101/gad.399506 16951251 PMC1560409

[17] Ribeiro de Almeida C Stadhouders R de Bruijn MJ Bergen IM Thongjuea S Lenhard B The DNA-binding protein CTCF limits proximal Vkappa recombination and restricts kappa enhancer interactions to the immunoglobulin kappa light chain locus. Immunity (2011) 35:501–13. 10.1016/j.immuni.2011.07.014 22035845

[18] Degner SC Verma-Gaur J Wong TP Bossen C Iverson GM Torkamani A CCCTC-binding factor (CTCF) and cohesin influence the genomic architecture of the Igh locus and antisense transcription in pro-B cells. Proc Natl Acad Sci U S A (2011) 108:9566–71. 10.1073/pnas.1019391108 21606361 PMC3111298

[19] Guo C Yoon HS Franklin A Jain S Ebert A Cheng HL CTCF-binding elements mediate control of V(D)J recombination. Nature (2011) 477:424–30. 10.1038/nature10495 21909113 PMC3342812

[20] Lin SG Guo C Su A Zhang Y Alt FW. CTCF-binding elements 1 and 2 in the Igh intergenic control region cooperatively regulate V(D)J recombination. Proc Natl Acad Sci U S A (2015) 112:1815–20. 10.1073/pnas.1424936112 25624508 PMC4330762

[21] Hu J Zhang Y Zhao L Frock RL Du Z Meyers RM Chromosomal loop domains direct the recombination of antigen receptor genes. Cell (2015) 163:947–59. 10.1016/j.cell.2015.10.016 26593423 PMC4660266

[22] Perez-Garcia A Marina-Zarate E Alvarez-Prado AF Ligos JM Galjart N Ramiro AR. CTCF orchestrates the germinal centre transcriptional program and prevents premature plasma cell differentiation. Nat Commun (2017) 8:16067. 10.1038/ncomms16067 28677680 PMC5504274

[23] Birshtein BK. The role of CTCF binding sites in the 3’ immunoglobulin heavy chain regulatory region. Front Genet (2012) 3:251. 10.3389/fgene.2012.00251 23162572 PMC3499808

[24] Pinaud E Marquet M Fiancette R Peron S Vincent-Fabert C Denizot Y The IgH locus 3’ regulatory region: pulling the strings from behind. Adv Immunol (2011) 110:27–70. 10.1016/B978-0-12-387663-8.00002-8 21762815

[25] Dunnick WA Shi J Graves KA Collins JT. The 3’ end of the heavy chain constant region locus enhances germline transcription and switch recombination of the four gamma genes. J Exp Med (2005) 201:1459–66. 10.1084/jem.20041988 15851486 PMC2213191

[26] Pinaud E Khamlichi AA Le Morvan C Drouet M Nalesso V Le Bert M Localization of the 3’ IgH locus elements that effect long-distance regulation of class switch recombination. Immunity (2001) 15:187–99. 10.1016/S1074-7613(01)00181-9 11520455

[27] Vincent-Fabert C Fiancette R Pinaud E Truffinet V Cogne N Cogne M Genomic deletion of the whole IgH 3’ regulatory region (hs3a, hs1,2, hs3b, and hs4) dramatically affects class switch recombination and Ig secretion to all isotypes. Blood (2010) 116:1895–8. 10.1182/blood-2010-01-264689 20538806

[28] Volpi SA Verma-Gaur J Hassan R Ju Z Roa S Chatterjee S Germline deletion of Igh 3’ regulatory region elements hs 5, 6, 7 (hs5-7) affects B cell-specific regulation, rearrangement, and insulation of the Igh locus. J Immunol (2012) 188:2556–66. 10.4049/jimmunol.1102763 22345664 PMC3430471

[29] Braikia F-Z Oudinet C Haddad D Oruc Z Orlando D Dauba A Inducible CTCF insulator delays the IgH 3’ regulatory region-mediated activation of germline promoters and alters class switching. Proc Natl Acad Sci U S A (2017) 114(23):6092–7. 10.1073/pnas.1701631114 28533409 PMC5468671

[30] Heath H Ribeiro de Almeida C Sleutels F Dingjan G van de Nobelen S Jonkers I CTCF regulates cell cycle progression of alphabeta T cells in the thymus. EMBO J (2008) 27:2839–50. 10.1038/emboj.2008.214 18923423 PMC2580790

[31] Rickert RC Roes J Rajewsky K. B lymphocyte-specific, Cre-mediated mutagenesis in mice. Nucleic Acids Res (1997) 25:1317–8. 10.1093/nar/25.6.1317 9092650 PMC146582

[32] Perez-Duran P Belver L de Yebenes VG Delgado P Pisano DG Ramiro AR. UNG shapes the specificity of AID-induced somatic hypermutation. J Exp Med (2012) 209:1379–89. 10.1084/jem.20112253 22665573 PMC3405504

[33] Reina-San-Martin B Difilippantonio S Hanitsch L Masilamani RF Nussenzweig A Nussenzweig MC. H2AX is required for recombination between immunoglobulin switch regions but not for intra-switch region recombination or somatic hypermutation. J Exp Med (2003) 197:1767–78. 10.1084/jem.20030569 12810694 PMC2193951

[34] Sernandez IV de Yebenes VG Dorsett Y Ramiro AR. Haploinsufficiency of activation-induced deaminase for antibody diversification and chromosome translocations both in vitro and in vivo. PLoS One (2008) 3:e3927. 10.1371/journal.pone.0003927 19079594 PMC2592691

[35] Nagaoka H Muramatsu M Yamamura N Kinoshita K Honjo T. Activation-induced deaminase (AID)-directed hypermutation in the immunoglobulin Smu region: implication of AID involvement in a common step of class switch recombination and somatic hypermutation. J Exp Med (2002) 195:529–34. 10.1084/jem.20012144 11854365 PMC2193625

[36] Schrader CE Bradley SP Vardo J Mochegova SN Flanagan E Stavnezer J. Mutations occur in the Ig Smu region but rarely in Sgamma regions prior to class switch recombination. EMBO J (2003) 22:5893–903. 10.1093/emboj/cdg550 14592986 PMC275407

[37] Xue K Rada C Neuberger MS. The in vivo pattern of AID targeting to immunoglobulin switch regions deduced from mutation spectra in msh2−/− ung−/− mice. J Exp Med (2006) 203:2085–94. 10.1084/jem.20061067 16894013 PMC2118391

[38] Rogozin IB Kolchanov NA. Somatic hypermutagenesis in immunoglobulin genes. II. Influence of neighbouring base sequences on mutagenesis. Biochim Biophys Acta (1992) 1171:11–8. 10.1016/0167-4781(92)90134-L 1420357

[39] Thomas-Claudepierre A-S Schiavo E Heyer V Fournier M Page A Robert I The cohesin complex regulates immunoglobulin class switch recombination. J Exp Med (2013) 210:2495–502. 10.1084/jem.20130166 24145512 PMC3832931

[40] Wuerffel R Wang L Grigera F Manis J Selsing E Perlot T S-S synapsis during class switch recombination is promoted by distantly located transcriptional elements and activation-induced deaminase. Immunity (2007) 27:711–22. 10.1016/j.immuni.2007.09.007 17980632 PMC4979535

